# Adenosine Downregulates the Activities of Glutamatergic Neurons in the Paraventricular Hypothalamic Nucleus Required for Sleep

**DOI:** 10.3389/fnins.2022.907155

**Published:** 2022-06-13

**Authors:** Changlin Chen, Yichen Lin, Feng Cai, Jinsui Li, Haixun Li, Xiantao Li

**Affiliations:** ^1^Department of Anesthesiology, The Affiliated Hospital of North Sichuan Medical College, Nanchong, China; ^2^Department of Vascular Surgery, The First Affiliated Hospital of Fujian Medical University, Fuzhou, China; ^3^Department of Urology and Neurocardiothoracic Surgery, 927 Hospital of the Joint Logistics Support Force of the Chinese People’s Liberation Army, Pu’er, China; ^4^Department of Thyroid and Breast Surgery, The Affiliated Hospital of North Sichuan Medical College, Nanchong, China; ^5^Department of Cardiovascular Surgery, The Third Affiliated Hospital of Zunyi Medical University, Zunyi, China

**Keywords:** sleep, wakefulness, adenosine, PVH, paraventricular nucleus of the hypothalamus, HCN channel

## Abstract

Adenosine is an endogenous substance that regulates sleep homeostasis. It plays an important role in sleep induction under physiological condition. So far, the neural mechanisms underlying sleep-promoting effects of adenosine are not completely clear. Recent studies have shown that glutamatergic neurons in the paraventricular hypothalamic nucleus (PVH) play an important role in wakefulness. Using whole-cell patch-clamp, we found that adenosine can inhibit glutamatergic neurons in PVH. This inhibition is mainly achieved by activating adenosine type 1 receptors, thereby reducing hyperpolarization-activated cyclic nucleotide-gated cation channels. By recording electroencephalogram (EEG) and electromyography (EMG), it was found that local administration of adenosine type 1 receptor blocker in PVH could significantly reduce the NREM sleep. On the contrary, if adenosine was given, it could increase the NREM sleep. These results suggest that adenosine can promote sleep by reducing the excitability of PVH neurons. This findings reveal a novel mechanism of adenosine regulating sleep homeostasis.

## Introduction

Adenosine is a metabolite after the decomposition of energy donor ATP. With the extension of awakening time, the concentration of adenosine in the brain would gradually increase. High concentrations of adenosine play a role in promoting sleep, and promote animals’ transitions from awakening to sleep. For this reason, adenosine is also known as a sleep homeostasis factor (Jones, 2009; Lazarus et al., 2019; Peng et al., 2020). How does adenosine promote sleep? It has been reported that there are mainly two ways. On the one hand, adenosine can directly inhibit the activities of cortical and subcortical arousal-promoting nuclei. For example, adenosine can directly inhibit the prefrontal cortex, and subcortical basal forebrain, lateral hypothalamus and other arousal-promoting brain areas (Liu and Gao, 2007; Sims et al., 2013; Cun et al., 2014; Peng et al., 2020). On the other hand, adenosine can excite some sleep-promoting brain regions, for example, adenosine can excite sleep-promoting neurons of ventral lateral preoptic area (VLPO) (Kumar et al., 2013). Although these studies have been reported, the mechanisms underlying the sleep-promoting effect of adenosine is not completely clear.

Recent studies have shown that glutamatergic neurons in paraventricular hypothalamic nucleus (PVH) play an important role in promoting arousal (Chen et al., 2021). These cells showed high-frequency discharge during awakening, and their activity decreased after awakening into sleep. The activation of these cells can significantly promote the transitions from sleep to awakening and prolong the awakening time. On the contrary, specific inhibition of these cells promotes the transitions from awakening to sleep (Liu et al., 2020; Chen et al., 2021).

In view of the important role of PVH in the regulation of arousal and sleep, we speculated that PVH may be an important target of adenosine. Using whole-cell patch-clamp recording and immunohistochemical techniques, we found that adenosine can significantly inhibit the activity of PVH glutamatergic neurons through adenosine type 1 receptors (A_1_Rs). *In vivo* studies have shown that the inhibitory effect of adenosine on PVH can promote sleep. These findings further reveal the neural mechanism of adenosine’s sleep promoting effect.

## Materials and Methods

### Preparation of Brain Slice

The experimental protocols involving mice were in line with the guidelines for the care and use of laboratory animals. Adult (8–12 weeks) vGlut2-Cre mice obtained from the Jackson lab (No. 016963). vGlut2-Cre mice were crossed with tdTomato reporter mice to label the glutamatergic neurons with the tdTomato in PVH. These adult transgenic mice were used to prepare the PVH slices. The coronal 400 μm slices containing the PVH were cut by a vibratome (7000SMZ, Campden). The brain slice were prepared in a cutting solution containing 110 mM NMDG, 110 mM HCl, 2.5 mM KCl, 1.2 mM NaH_2_PO_4_, 25 mM NaHCO_3_, 25 mM Glucose, 10 mM MgSO_4_, 0.5 mM CaCl_2_ with 95% O_2_ and 5% CO_2_ saturation. pH of this cutting solution was adjusted to ∼7.25. After that, the brain slices containing PVH were transferred to artificial cerebrospinal fluid (ACSF) which contained 124 mM NaCl, 3 mM KCl, 26 mM NaHCO_3_, 2 mM MgCl_2_, 2 mM CaCl_2_ and 10 mM glucose with oxygen (95% O_2_–5% CO_2_) saturation for at least 40 min. During recording sessions, the slices were removed to a chamber with continuous superfusion of ACSF at ∼32°C.

### Patch-Clamp Recordings

The targeted neurons expressing tdTomato were identified by a fluorescence microscope (Olympus, Japan). The glass pipettes filled with a recording solution (125 mM potassium gluconate, 20 mM KCl, 10 mM HEPES, 1 mM EGTA, 2 mM MgCl_2_, 4 mM ATP) with resistance of 3–5 MΩ were used for whole-cell patch-clamp recordings. For current clamp model, the membrane potentials were held at −60 mV. Signals were acquired after at least 5 min formation of whole-cell configuration. We compensated the series resistance about 75%. Cells were discarded from analyses if the series resistance exceeded 20 MΩ or increased by >25% during recording sessions. Signals were collected by EPC10 amplifier (HEKA Elektronik, Germany). The signals were low-pass-filtered at with cut-off frequency of 4 kHz. The off-line data were analyzed by the Pulsefit and Igor Pro (WaveMetrics) software.

### Single Cell Reverse Transcription PCR (scRT-PCR)

After whole cell recording, cytoplasm was sucked into glass pipettes containing internal solution. The PrimeScript™ II 1st Strand cDNA Synthesis Kit (6210A, TaKaRa) was used to prepare the cDNA of each cell. Briefly, the reaction mixture was cooled on ice following denaturing at 65°C for 5 min. After that, the Oligo dT primer and random primers were added to the reaction mixture. The reaction conditions were performed at 45°C for 60 min and then at 70°C for 15 min. The PCR amplification was performed with the gene-specific multiplex primers using the SuperScript III One-Step RT-PCR Kit (12574026, Invitrogen). cDNA synthesized in the reverse transcription step was made as the template for the first round of PCR amplification, the reaction was performed as follows: 2 min at 95°C; 40 cycles of 15 s at 94°C, 30 s at 58°C, and 30 s at 67°C; 5 min at 67°C. Next, PCR was carried out with nested primers for each gene and the first PCR product was used as the template. The second reaction was performed as follows: 2 min at 95°C; 45 cycles of 15 s at 94°C, 30 s at 56°C, and 30 s at 67°C; 5 min at 67°C. Finally, the amplification products were visualized by electrophoresis in 2% agarose gels. Primers (5′-3′) for single-cell RT-PCR: β*-actin* (sense/anti-sense): multiplex, 5′ gacccagatcatgtttgagacc/gctaggagccagagcagtaatct; nested, aggctgtgctgtccctgtatg/gaggtctttacggatgtcaacg Final product 470 bp *Slc17a6* (encoding VGLUT2) (sense/anti-sense): multiplex, 5′ tgttctggcttctggtgtcttacgagag/ttcccgacagcgtgccaaca; nested, tcaacaacagcaccatccac/gggctctcgtaagacaccag. Final product 315 bp *Gad1* (sense/anti-sense): multiplex, cacaggtc accctcgatttt/tctatgccgctgagtttgtg; nested, tagctggtgaatggctgaca/cttgtaacgagcagccatga. Final product 200 bp.

### Drugs

Adenosine, DPCPX, and ZD7288 were obtained from Sigma. These drugs were prepared as stock solutions and frozen at −25°C. The drugs from stock were diluted to the final concentrations until use. To investigate the effects of the adenosine on membrane properties and whole cell currents, adenosine was bathed to the slices for 2 min.

### Electroencephalogram/Electromyography Electrodes and Cannula Implantation

The mice were anesthetized with isoflurane (2%), placed in the brain stereotactic instrument, disinfected with alcohol in turn, removed the soft tissue on the surface of the skull. Drill holes in frontal lobe (AP: +1.0 mm, ML: ±1.5 mm) and right parietal lobe (AP: −2.0 mm, ML: ±1.5 mm). Stainless steel screws (diameter 1 mm) for collecting electroencephalogram (EEG) electrodes were fixed in the drilling hole of the fabricated skull, and two electrodes for collecting electromyography (EMG) were buried in the neck muscle.

According to the positioning coordinates of the PVH (AP = −0.5 mm; ML = ± 0.2 mm; DV = −4.2 mm), the bone hole for implantation the cannula was made on the surface of the skull with a dental drill, and the cannula was pushed to the PVH with a microdriver. The fixing screw, EEG/EMG recording electrodes and cannula were fixed on the surface of skull with dental cement. After the operation, the mice were placed in the cage for recovery. After 7 days of recovery, EEG/EMG monitoring was carried out.

### Drug Microinjection

Microinjection pump (IVM-1000, Scientifica, United Kingdom) was used for drug microinjection. According to a previous study (Chen et al., 2021), we chose the administration volume 100 nl. The microinjection rate was 50 nl per min. In order to fully diffuse the drug, the injection needle was kept in place for 1 min after administration. After administration, the animals were immediately connected to the recording system for EEG/EMG monitoring.

### Electroencephalogram/Electromyography Data Acquisition and Analysis

The mice were connected with the recording line through the universal runner. During recording, the room temperature was kept at 22–23°C, with alternating light and dark, light: 8:00–20:00; Darkness: 20:00–8:00). After the mice adapted to the recording scene, the data of 1 day were recorded to evaluate the quality of EEG/EMG and whether the sleep/wake rhythm of the mice was normal.

After the mice adapted to the recording system, the microinjection was performed. In order to eliminate the influence of sleep rhythm, the administration time was fixed at 8:00 or 20:00. The obtained data were analyzed by the professional software (SleepSign for animals, Kissei Comtec), and the corresponding state of EEG/EMG was analyzed. Brain states were classified by a consecutive non-overlapping 6 s epoch. Wakefulness was defined as low-amplitude and desynchronized EEG and enhanced EMG activity. NREM sleep was defined as high-amplitude and low frequency EEG activity, and deceased EMG activity. REM sleep was defined as containing a pronounced theta (4–12 Hz) rhythm with nearly no EMG activity (Ren et al., 2018). EEG power spectrum was analysed by SleepSign software. The absolute EEG power of each 0.25 Hz bin was normalized to the sum of the power over the entire analysis range (0–25 Hz) of the same state. Relative bins were summed in delta (0.25–4 Hz) and theta (4–10 Hz) bands.

### Immunohistochemistry and Injection Site Detection

To label the glutamatergic neurons with the tdTomato, vGlut2-Cre mice were crossed with tdTomato reporter mice. These hybrid mice were anesthetized with isoflurane, perfused transcardially with 0.9% saline, and followed by 4% paraformaldehyde (PFA, 0–4°C) in PBS. Brains were fixed in 4% PFA for another 12 h, dehydrated with 30% sucrose (4°C, 24 h), and the brain sections were prepared with a freezing microtome (CM 3050S, Leica).

For immunohistochemical staining, brain slices were washed by 0.01 M PBS, removed to a blocking solution (0.1% Triton X-100, 5% normal donkey serum) at 37°C for 30 min, and then incubated with primary antibody (abcam, ab223377) in blocking solution at 4°C for 24 h. After that, sections were washed in 0.01 M PBS 3 times, moved into secondary antibody (568 donkey anti-rabbit, 1:1,000, Invitrogen) in blocking solution and incubated for 2 h at room temperature. Finally, brain slices were mounted on glass slides, and cover-slipped using Fluoroshield with DAPI (Sigma-Aldrich). These sections were observed and the images were obtained by an LSM 780 confocal microscope (Carl Zeiss, Jena, Germany).

After behavioral experiments, the mice were perfused with 0.9% NaCl (37°C) and 4% paraformaldehyde (0–4°C), and the brain was removed immediately after perfusion. It was fixed in 4% paraformaldehyde and then transferred to 30% sucrose and 4% paraformaldehyde solution for dehydration. After the brain tissue sinked to the bottom, freeze brain sections with 50 μm thickness were prepared. The PVH was sectioned consecutively according to the coronal plane, stained with DAPI, and the cannula track was observed under the fluorescence microscope. Animals with inaccurate injection site were not included in the analysis.

### Data Analysis

Data were presented as the means ± SEM. One sample *t*-test, Student’s paired *t*-test, one-way repeated-measures’ analysis of variance, and Fisher’s protected least significant difference *post hoc* testing were performed for statistical analyses. If data did not conform to the normal distribution, we performed non-parametric tests. The statistical significant differences were accepted when *P* < 0.05.

## Results

### Adenosine Inhibits the Excitability of the Glutamatergic Neurons in the Paraventricular Hypothalamic Nucleus via Stimulating Adenosine Type 1 Receptors

**FIGURE 1:**
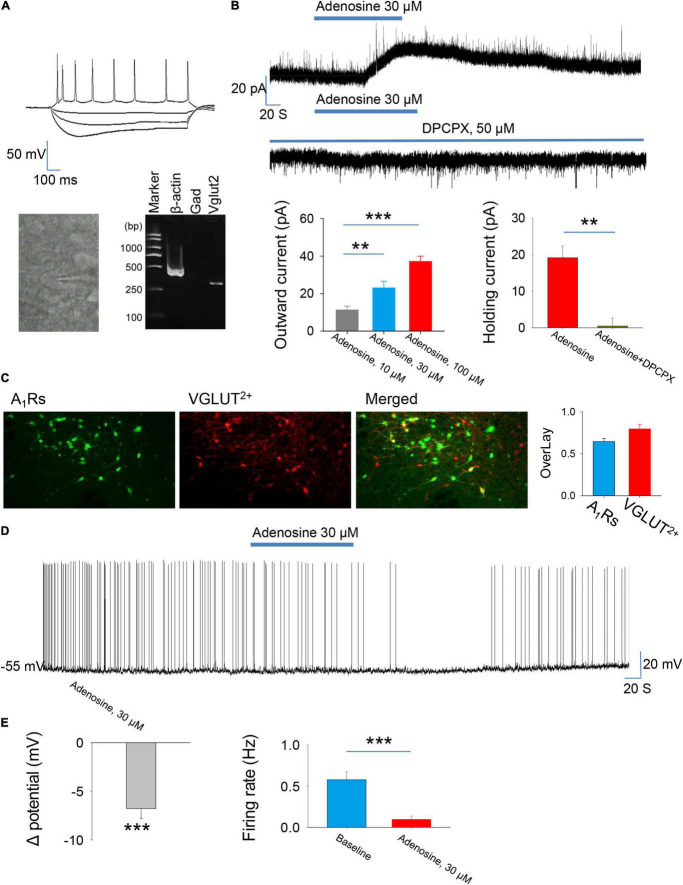
Adenosine reduces the activity of glutamatergic neurons in the paraventricular hypothalamic nucleus (PVH) via adenosine 1 receptors (A_1_Rs). **(A)** Top, the morphology and the voltage responses induced by inward current injection of a recorded glutamatergic neuron in the PVH. Bottom, single-cell reverse transcription-polymerase chain reaction (scRT-PCR) showed that the recorded neurons were VGLUT2^+^. **(B)** Adenosine induced outward currents in a concentration-dependent manner (*n* = 8 cells, one-way repeated-measures’ analysis of variance, and Fisher’s protected least significant difference post hoc testing, F_(2,23)_ = 19.007, *P* < 0.001). Adenosine-induced outward currents were completely blocked by the A1R antagonist DPCPX (*n* = 6 cells, t_5_ = 5.47, Paired *t*-test, *P* < 0.01). **(C)** Left, the images show the expression of A_1_Rs in the glutamatergic neurons in the PVH. Right, the percentage of overlay neurons colocalized with A_1_Rs and VGLUT2. **(D,E)** Adenosine elicited membrane hyperpolarization and reduced the firing rate. Membrane potentials: *n* = 9 cells, t_8_ = –6.62, One-Sample *t*-test, *P* < 0.001; Firing rates: *n* = 9 cells, t_8_ = 5.588, Paired *t*-test, *P* < 0.001.

We first wanted to know the effect of adenosine on the glutamatergic neurons in the PVH. The glutamatergic neurons expressing tdTomato were specifically detected from the brain slices. Consistently, scRT-PCR combined with whole-cell patch clamp showed that the identified neurons (n = 5) were positive for VGLUT2, but not GAD1, indicating that these neurons were glutamatergic (Figure 1A). Voltage-clamp recordings were carried out to test the effects of adenosine on the whole-cell currents. Bath application (10–100 μM) of adenosine produced outward currents in all the recorded neurons. The amplitude of outward currents caused by adenosine was concentration-dependent (Figure 1B).

Then, we wanted to know the receptor mechanism of adenosine-induced outward currents. We found that the effect of adenosine was almost completely blocked in the presence of A_1_R blocker, DPCPX (50 μM) (Figure 1B). Furthermore, immunohistochemistry showed that there was a high abundance of A_1_R expression in PVH glutamate neurons (Figures 1C). These results suggest that adenosine plays a role mainly by activating A_1_Rs.

Under current-clamp mode, adenosine (30 μm) remarkably hyperpolarized the PVH glutamatergic neurons, and significantly suppressed the firing rates of these neurons (Figures 1D,E), suggesting an inhibitory effect of adenosine on the PVH glutamatergic neurons.

### The Downregulation of Hyperpolarization-Activated Cyclic Nucleotide-Gated Channels Mediates the Inhibitory Effects of Adenosine

Then, we explored the ion channel mechanisms underlying adenosine-induced inhibition. A_1_R activation stimulates the downstream cAMP-PKA pathway, which has been reported to regulate hyperpolarization-activated cyclic nucleotide-gated cation (HCN) channels (Li et al., 2011; Garcia-Gil et al., 2021). Thus, we hypothesized that changes in HCN channel activity might mediate the inhibitory effect of adenosine.

**FIGURE 2:**
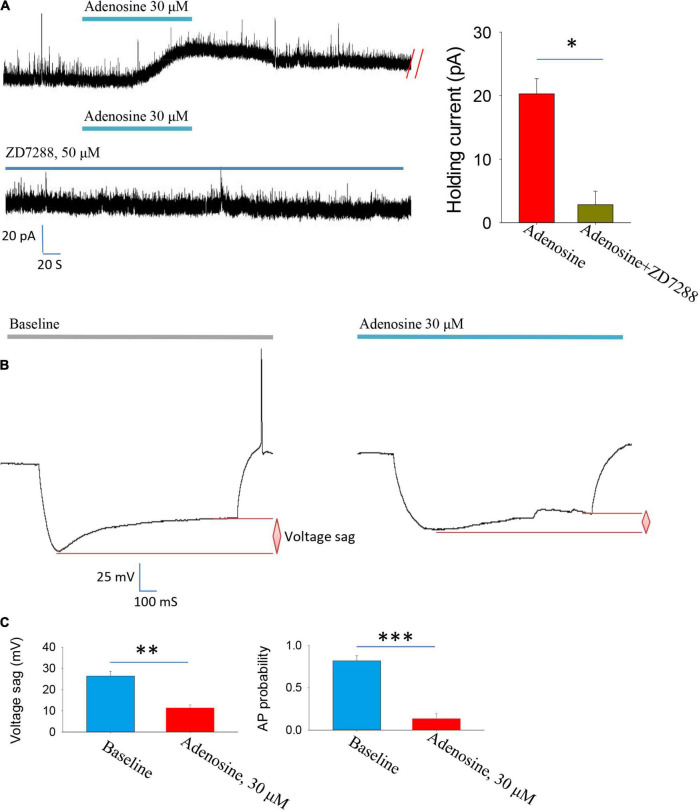
Adenosine reduces the activity of glutamatergic neurons in the PVH *via* downregulating Ih currents. **(A)** Adenosine-induced outward currents were abolished by the HCN channel antagonist ZD7288. *n* = 7 cells, Z = −2.371, Wilcoxon Signed Rank Test, *P* < 0.05. **(B,C)** Application of adenosine reduced the HCN channels-related voltage sag and rebound firing frequency. AP, action potentials. Voltage sag: *n* = 6 cells, t_5_ = 5.26, Paired *t*-test, *P* < 0.01; AP probability: *n* = 6 cells, t_5_ = 7.83, Paired *t*-test, *P* < 0.001.

To test this hypothesis, we used HCN channel blocker ZD7288 and found that the outward currents induced by adenosine were completely blocked in the presence of this blocker (Figure 2A). Membrane hyperpolarization could activate HCN channels, which in turn induces depolarization and induced voltage sags. We then directly recorded the HCN channel activation-induced voltage sags. Indeed, application of adenosine significantly inhibited HCN channel activation-induced voltage sags. Additionally, HCN channels-induced rebound firing was dramatically reduced by adenosine (Figures 2B,C). Altogether, these results suggest that adenosine downregulate HCN channels to inhibit the PVH glutamatergic neurons.

### Blockade of Adenosine Type 1 Receptors Reduces NREM Sleep During the Light Phase

**FIGURE 3:**
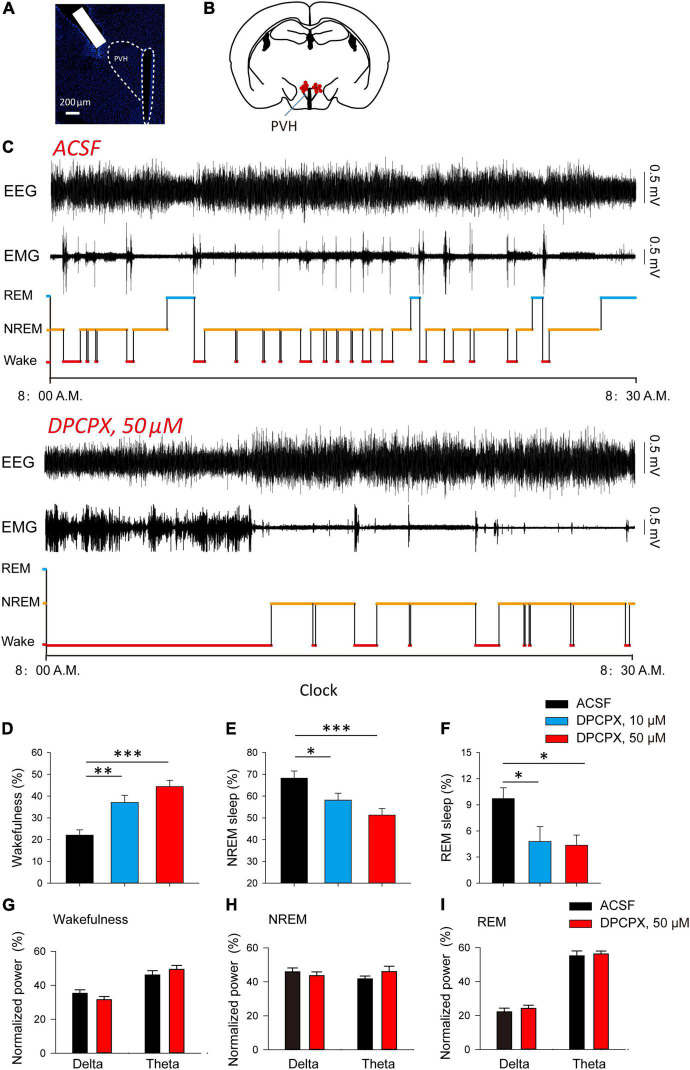
Blockade of A_1_Rs increases wakefulness during light phase. **(A)** DAPI staining showed the location of cannula. **(B)** The microinjection sites of the recorded mice included in the behavioral analyses. **(C)** Reprehensive EEG, EMG and hypnogram during 0.5 h post-vehicle, or A_1_Rs antagonist injection, into PVH during the light phase. **(D–F)** Quantitative analysis of time spent in NREM, wake and REM states. Wakefulness: *n* = 7 mice, one-way repeated-measures’ analysis of variance, and Fisher’s protected least significant difference post hoc testing, F(2,20) = 15.590, *P* < 0.001; NREM: *n* = 7 mice, one-way repeated-measures’ analysis of variance, and Fisher’s protected least significant difference post hoc testing, F_(2,20)_ = 7.211, *P* < 0.01; REM: *n* = 7 mice, one-way repeated-measures’ analysis of variance, and Fisher’s protected least significant difference post hoc testing, F_(2,20)_ = 4.528, *P* < 0.05. **(G–I)** The power spectrum of EEG recorded during wakefulness **(G)**, NREM **(H)** and REM sleep **(I)**, respectively. Wakefulness (delta: *n* = 7 mice, t_6_ = 1.81, Paired *t*-test, *P* < 0.12; theta: *n* = 7 mice, t_6_ = 1.87, Paired *t*-test, *P* < 0.11); NREM (delta: *n* = 7 mice, t_6_ = 0.79, Paired *t*-test, *P* < 0.46; theta: *n* = 7 mice, t_6_ = −1.82, Paired *t*-test, *P* < 0.12); REM (delta: *n* = 7 mice, t_6_ = −0.82, Paired *t*-test, *P* < 0.44; theta: *n* = 7 mice, t_6_ = −0.70, Paired *t*-test, *P* < 0.51).

After that, we explored whether the inhibitory effect of adenosine can affect the awakening and sleep behavior related to PVH glutamatergic neurons. We implanted cannula in the PVH to analyze the changes in sleep/wake time after administration of different concentrations of A_1_R blocker, DPCPX (Figures 3A,B). We found that application of DPCPX significantly reduced NREM and REM sleep time, and promoted wakefulness in a concentration-dependent manner (Figures 3D–F). However, application of DPCPX did not affect the power spectrum of EEG recorded during wakefulness, NREM and REM sleep, respectively (Figures 3G–I). The results suggested that the A_1_R activation in the PVH play an important role in sleep induction under physiological conditions.

### Adenosine Type 1 Receptor Activation by Local Application of Adenosine During Dark Phase Promote Sleep

**FIGURE 4:**
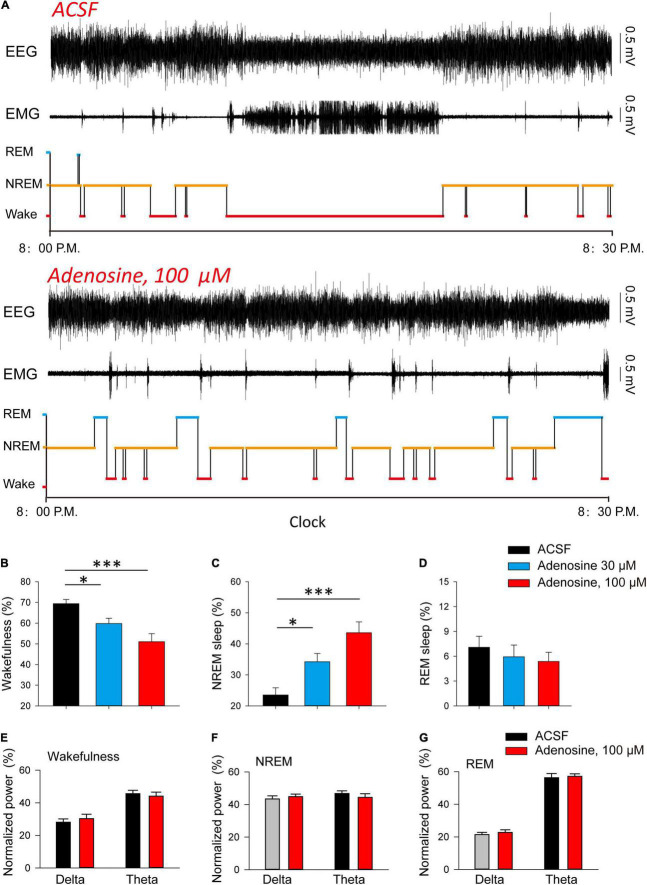
Injection of adenosine in the PVH increases NREM sleep during dark phase. **(A)** Reprehensive hypnogram during 0.5 h post-vehicle, or adenosine injection, into PVH during the dark phase. **(B–D)** Quantitative analysis of time spent in NREM, wake and REM states. Wakefulness: *n* = 10 mice, one-way repeated-measures’ analysis of variance, and Fisher’s protected least significant difference post hoc testing, F_(2,29)_ = 8.775, *P* < 0.01; NREM: *n* = 10 mice, one-way repeated-measures’ analysis of variance, and Fisher’s protected least significant difference post hoc testing, F_(2,29)_ = 12.2, *P* < 0.001; REM: *n* = 10 mice, Kruskal-Wallis One Way Analysis of Variance on Ranks, H_2_ = 0.839, *P* < 0.657. **(E–G)** The power spectrum of EEG recorded during wakefulness **(E)**, NREM **(F)** and REM sleep **(G)**, respectively. Wakefulness (delta: *n* = 10 mice, t_9_ = −1.00, Paired *t*-test, *P* < 0.34; theta: *n* = 10 mice, t_9_ = 0.90, Paired *t*-test, *P* < 0.39); NREM (delta: *n* = 10 mice, t_9_ = −0.75, Paired *t*-test, *P* < 0.47; theta: *n* = 10 mice, t_9_ = 0.86, Paired *t*-test, *P* < 0.41); REM (delta: *n* = 10 mice, t_9_ = −0.57, Paired *t*-test, *P* < 0.58; theta: *n* = 10 mice, t_9_ = −0.51, Paired *t*-test, *P* < 0.62).

Finally, we investigated whether adenosine is sufficient to induce sleep. We found that microinjection of adenosine into PVH could significantly reduce the awakening time, and increase NREM sleep time, but had no significant effect on REM sleep (Figures 4A–D). Application of adenosine did not affect the power spectrum of EEG recorded during wakefulness, NREM and REM sleep, respectively (Figures 4E–G). These results suggested that adenosine-induced inhibition of PVH glutamatergic neurons could promote sleep.

## Discussion

The occurrence of sleep is mainly regulated by rhythmic and homeostatic factors. Adenosine is an important factor to realize the regulation of sleep homeostasis (Jones, 2009; Lazarus et al., 2019; Peng et al., 2020). In this study, we found that adenosine can down regulate the activity of HCN channel by activating A_1_Rs, and reduce the excitability of glutamatergic neurons in the PVH. This effect of adenosine is involved in the regulation of sleep under physiological conditions, because the inhibition of adenosine receptor in the PVH can significantly reduce the occurrence of sleep. These findings further reveal the neural mechanism underlying the homeostatic regulation of sleep by adenosine.

The wakefulness/sleep behavior is regulated by multiple subcortical nuclei in the brain. Wakefulness depends on the acetylcholinergic neurons in basal forebrain, the norepinephrine neurons in locus coeruleus, the serotonin neurons in dorsal raphe nucleus, the glutamatergic neurons in parabrachial nucleus, the dopaminergic neurons in ventral tegmental area, the histaminergic, orexinergic, and γ-aminobutyric neurons in hypothalamus, dopamine D1 receptor positive neurons in the nucleus accumbens, and glutamatergic neurons in the midline nucleus of thalamus. NREM sleep is controlled by GABA neurons in preoptic area of hypothalamus, parafacial area of brain stem and reticular nucleus of thalamus. The increased functional activity of sleep-promoting nuclei accompanied by a decreased arousal-promoting neuronal activity induces the transitions from arousal to sleep (Lee and Dan, 2012; Xu et al., 2015; Chung et al., 2017; Chen et al., 2018; Liu and Dan, 2019; Zhang et al., 2019). Recently, many studies have reported that PVH is an important arousal-promoting nucleus (Liu et al., 2020; Chen et al., 2021). If PVH is damaged, it can cause serious symptoms such as drowsiness. PVH contains many types of neurons, including glutamatergic neurons and GABAergic neurons. Using cell specific labeling and manipulation technologies, it was found that glutamate cells in PVH showed a high level of discharge during awakening, while the discharge frequency decreased when NREM sleep approaching. Using optogenetic and chemogenetic manipulation techniques, it was further found that specific activation of these cells can strongly increase the awakening time. On the contrary, inhibition of these cells promotes NREM sleep (Liu et al., 2020; Chen et al., 2021). However, it is unclear how the activity of PVH glutamate neurons is regulated. We found that adenosine can inhibit the activity of PVH glutamate neurons, and then induced the transitions from arousal to sleep. This discovery reveals a new mechanism of PVH regulating wakefulness/sleep behavior.

Adenosine has long been reported to be involved in sleep (Jones, 2009; Lazarus et al., 2019). Although adenosine has a strong sleep-promoting effect, its mechanism is not completely clear. At present, it is believed that adenosine plays a role by activating sleep-promoting brain areas and inhibiting arousal-promoting brain areas, such as acetylcholinergic neurons in the basal forebrain and orexinergic neurons in the lateral hypothalamus (Thakkar et al., 2008; Lazarus et al., 2019; Peng et al., 2020). This study found that adenosine can inhibit PVH, another important arousal-promoting nucleus, and revealed the receptor and ionic mechanisms underlying its inhibition. This discovery further revealed the neural mechanism of adenosine regulating sleep and expanded our understanding of the mechanism of sleep induction.

## Data Availability Statement

The raw data supporting the conclusions of this article will be made available by the authors, without undue reservation.

## Ethics Statement

The animal study was reviewed and approved by The Affiliated Hospital of North Sichuan Medical College, Nanchong.

## Author Contributions

CC and YL conducted the whole-cell patch-clamp recordings. FC, JL, HL, and XL performed the behavioral experiments. All authors contributed to the article and approved the submitted version.

## Conflict of Interest

The authors declare that the research was conducted in the absence of any commercial or financial relationships that could be construed as a potential conflict of interest.

## Publisher’s Note

All claims expressed in this article are solely those of the authors and do not necessarily represent those of their affiliated organizations, or those of the publisher, the editors and the reviewers. Any product that may be evaluated in this article, or claim that may be made by its manufacturer, is not guaranteed or endorsed by the publisher.
